# Clinical and epidemiological profiles from a case series of 26 Brazilian CADASIL patients

**DOI:** 10.1055/s-0042-1758756

**Published:** 2023-05-08

**Authors:** Renata Nogueira, Christian Marques Couto, Pérola de Oliveira, Bernardo José Alves Ferreira Martins, Vinícius Viana Abreu Montanaro

**Affiliations:** 1 Rede Sarah de Hospitais de Reabilitação, Rio de Janeiro RJ, Brazil.; 2 Rede Sarah de Hospitais de Reabilitação, Brasília DF, Brazil.

**Keywords:** CADASIL, Stroke, Epidemiology, Brazil, Receptor, Notch3, CADASIL, Acidente Vascular Cerebral, Epidemiologia, Brasil, Gene NOTCH3

## Abstract

**Background** Cerebral autosomal dominant arteriopathy with subcortical infarcts and leukoencephalopathy (CADASIL) is a genetic cause of ischemic stroke and the most common form of non-atherosclerotic stroke. Despite being the most prevalent vascular hereditary disease, clinical data regarding the Brazilian population are scarce. Considering that the Brazilian population has one of the most heterogeneous genetic constitutions in the world, knowledge about genetic and epidemiological profiles is mandatory. The present study aimed to elucidate the epidemiological and clinical features of CADASIL in Brazil.

**Methods** We performed a case series study comprising 6 rehabilitation hospitals in Brazil and reported the clinical and epidemiological data from the medical records of patients admitted from 2002 to 2019 with genetic confirmation.

**Results** We enrolled 26 (16 female) patients in whom mutations in exons 4 and 19 were the most common. The mean age at the onset of the disease was of 45 years. Ischemic stroke was the first cardinal symptom in 19 patients. Cognitive impairment, dementia, and psychiatric manifestations were detected in 17, 6, and 16 patients respectively. In total, 8 patients had recurrent migraines, with aura in 6 (75%) of them. White matter hyperintensities in the temporal lobe and the external capsule were found in 20 (91%) and 15 patients (68%) respectively. The median Fazekas score was of 2. Lacunar infarcts, microbleeds, and larger hemorrhages were observed in 18 (82%), 9, and 2 patients respectively.

**Conclusion** The present is the most extensive series of Brazilian CADASIL patients published to date, and we have reported the first case of microbleeds in the spinal cord of a CADASIL patient. Most of our clinical and epidemiological data are in accordance with European cohorts, except for microbleeds and hemorrhagic strokes, for which rates fall in between those of European and Asian cohorts.

## Resumo

**Antecedentes** Arteriopatia cerebral autossômica dominante com enfartes subcorticais e leucoencefalopatia ( *Cerebral Autosomal Dominant Arteriopathy with Subcortical Infarcts and Leukoencephalopathy* , CADASIL, em inglês) é uma causa genética de acidente vascular cerebral (AVC) isquêmico e a forma mais comum de acidente vascular cerebral não aterosclerótico. Apesar de ser a doença vascular hereditária mais prevalente que há, os dados clínicos para a população brasileira são escassos. Considerando que o Brasil tem uma das constituições genéticas mais heterogêneas do mundo, o conhecimento sobre perfis genéticos e epidemiológicos é obrigatório. Este estudo teve como objetivo elucidar as características clínicas e epidemiológicas de pacientes com CADASIL no Brasil.

**Métodos** Apresentamos uma série de casos envolvendo 6 hospitais de reabilitação no Brasil, e relatamos dados clínicos e epidemiológicos de prontuários de pacientes admitidos entre 2002 e 2019 com confirmação genética.

**Resultados** incluímos 26 pacientes (16 mulheres) em que as mutações nos éxons 4 e 19 eram as mais comuns. A idade média de início da doença foi de 45 anos. O AVC isquêmico foi o primeiro sintoma cardinal em 19 pacientes. Comprometimento cognitivo, demência e manifestações psiquiátricas foram detectados em 17, seis e 16 pacientes, respectivamente. Ao todo, 8 pacientes apresentavam enxaqueca, sendo com aura em 6 (75%) pacientes. Hiperintensidades de substância branca no polo temporal e na cápsula externa foram encontradas em 20 (91%) e 15 pacientes (68%), respectivamente. A pontuação mediana na escala de Fazekas foi de 2. Infartos lacunares, microssangramentos e macro-hemorragias foram observadas em 18 (82%), 9 (41%) e 2 (9%) pacientes, respectivamente.

**Conclusão** O presente estudo representa a mais extensa série de pacientes brasileiros com CADASIL publicada até o momento, e relatamos o primeiro caso de micro-hemorragia na medula espinhal de um paciente com CADASIL. A maior parte dos nossos dados clínicos e epidemiológicos está de acordo com as coortes europeias, exceto para micro-hemorragias e macro-hemorragias, para as quais as taxas se enquadram entre as das coortes europeias e asiáticas.

## INTRODUCTION

Cerebral autosomal dominant arteriopathy with subcortical infarcts and leukoencephalopathy (CADASIL) is the most prevalent hereditary monogenic vascular disease, with an estimated prevalence of 2 to 5 cases for every 100 thousand individuals. 1 It is caused by mutations in the *NOTCH3* gene located on chromosome 19, which leads to an uneven number of cysteine residues in a transmembrane receptor found in systemic and intracranial arterial smooth muscle cells. 2 3

Despite being the most prevalent vascular hereditary disease, clinical and epidemiological data are still emerging. A systematic review 4 published in 2020 detailed the clinical data of 752 patients with CADASIL, but most were from European or Asian countries. South American patients represented less than 1% of the series, 4 and Brazilian patients were not included. An Argentinian report 5 of 13 patients is the most extensive study in South America to date. Considering that the Brazilian population has one of the most heterogeneous genetic constitutions in the world, more data from these patients are necessary. 6 7

We aimed to describe the clinical and epidemiological aspects of all patients admitted to our hospital between 2002 and 2019 in all 6 hospitals of our rehabilitation network.

## METHODS

### Study design and participants

The present was a case series study comprising 6 rehabilitation hospitals from the same open-access quaternary network unit across Brazil. All patients were admitted between 2002 and 2019.

The inclusion criteria were admission between 2002 and 2019 and a molecular diagnosis of CADASIL. However, genetic testing was heterogeneous, and not all known exons were sequenced for every patient. In most cases, only exons 3 and 4 were analyzed because of technical limitations. General demographic information and clinical manifestations were obtained during the first medical visit.

The following information was recorded: sex, family history of CADASIL, presence of cardiovascular risk factors, age at onset of major clinical events, cardinal clinical features of CADASIL, neurologic examination findings, functional dependence as measured by the modified Rankin scale (mRs), and the use of anti-aggregation or anticoagulation. In addition, we also registered if a diagnosis of CADASIL had already been considered before admission, as well as all other differential diagnoses.

The relatives of the probands were included if they met the inclusion criteria. Family members were not actively recruited. The following cardiovascular risks were assessed: hypertension, hypercholesterolemia, smoking, diabetes mellitus, and alcoholism. The four cardinal symptoms of CADASIL are stroke/transient ischemic attack (TIA), cognitive dysfunction, migraine, or psychiatric symptoms. 1 Family history was considered positive when at least one cardinal symptom was present in one proband's first-degree relative.

Major clinical events were defined as stroke, encephalopathy, seizure, dementia, gait abnormalities, pseudobulbar palsy, and parkinsonian syndrome. Psychiatric symptoms and migraines were not considered the first symptom because these disorders are highly prevalent in the general population.

The *NOTCH3* gene was sequenced from blood DNA samples, in the forward and reverse directions, on an ABI PRISM 377 (Applied Biosystems, Foster City, CA, United States) DNA sequencer using the ABI PRISM BigDye Terminator Cycle Sequencing Ready Reaction Kit (Applied Biosystems). The samples in which a deletion was detected underwent a procedure aimed at separating the alleles to better show the mutation. The alleles were separated using electrophoresis on a 4% agarose gel. The alleles were then sequenced separately.

A long-term experienced neuroradiologist reviewed all brain magnetic resonance imaging (MRI) scans. MRI sequences analyzed included at least a T2, a diffusion, and a two-dimensional (2D) or three-dimensional (3D) fluid-attenuated inversion recover (FLAIR) axial images, and a sagittal T1 and T2 two-dimensional (2D) or three-dimensional (3D) images. Sequences were acquired with one of the following whole-body MRI scanners: Signa HDx 1,5T (G.E. Healthcare, Waukesha, WI, United States), Signa HDxT 1,5T (G.E. Healthcare), Signa HDxT 3T (G.E. Healthcare), Symphony 1,5T (Siemens Healthineers, Erlangen, Germany), Aera 1,5T (Siemens Healthineers), or Tim Trio 3T (Siemens Healthineers). The following aspects of the image were analyzed: presence and site of lacunar infarcts, presence and number of cerebral microbleeds (CMBs), the presence of white matter hyperintensities (WMHs) in the anterior temporal poles and external capsule, the severity of the WMHs according to the simplified Fazekas scale (range: 0–3), and the vascular load according to the total small vessel disease (SVD) score. 8 9

The SVD scores ranged from 0 to 4 points. It is calculated by adding a point for each of the findings: at least 1 lacune, at least 1 deep CMB, moderate to severe WHM (periventricular Fazekas-3 WMH and/or deep Fazekas 2-3 WMH), and moderate to severe (> 10) perivascular spaces in the basal ganglia. 9

The protocol of the present study followed resolution 466/12 of the Brazilian Ministry of Health, and it was approved by the ethics committee of the SARAH Network of Rehabilitation Hospitals. The need for patient consent was waived by the committee.

### Statistical analysis

The categorical data are presented as numbers and percentages, and the continuous data, as mean or median values. All statistical analyses were performed using the Statistical Package for the Social Sciences (IBM SPSS Statistics for Windows, IBM Corp., Armonk, NY, United States) software, version 23. The continuous variables were expressed as mean with standard deviation values for normally-distributed variables, and as median and interquartile range values for non-normally-distributed variables. The Kolmogorov-Smirnov test was used to assess the normality of the variables.

## RESULTS

We identified 26 individuals from 22 pedigrees with confirmed CADASIL. The mean age at assessment was of 51.7 years. There were 16 female (62%) and 10 male (38%) patients.

In total, 16 distinct mutations in the *NOTCH3* gene were identified, and mutations in exon 4 were the most common, representing 19 individuals of 17 pedigrees. Furthermore, mutations were found in exon 19 in 4 family members from 2 pedigrees, and mutations in exons 5, 11, and 18 were found in 1 individual ( Table 1 ). However, the mutation analysis of exons 2-23 from NOTCH3 was not performed in all patients, and eight patients had their mutation analysis limited to exons 3, 4, and 11. All mutations identified in the present study were previously described and considered pathogenic according to the American College of Medical Genetics and Genomics and the Association.

**Table 1:** Genetic profile of all patients included in the study

Family	Patients	Nucleotide change	Amino acid change	Exon	Exons sequenced
Family 1	Patients 1, 2, 3	c.3043T > C	p.1015Cys > Arg	19	Information not available
Family 2	Patients 4, 5	c.457C > T	p.153Arg > Cys	4	3 and 4
Family 3	Patients 6, 7	c.505C > T	p.169Arg > Cys	4	Information not available
Family 4	Patient 8	c.509A > G	p.170His > Arg	4	Information not available
Family 5	Patient 9	c.602G > A	p.201Cys > Tyr	4	3, 4 and 19
Family 6	Patient 10	c.509A > G	p.170His > Arg	4	Information not available
Family 7	Patient 11	c.397C > T	p.133Arg > Cys	4	Information not available
Family 8	Patient 12	c.2932A > C	p.978Ser > Arg	18	Information not available
Family 9	Patient 13	c.544C > T	p.182Arg >Cys	4	3 and 4
Family 10	Patient 14	c.397C > T	p.133Arg > Cys	4	3, 4 and 19
Family 11	Patient 15	c.457C > T	p.153Arg > Cys	4	3 and 4
Family 12	Patient 16	c.751T > A	p.251Cys > Ser	5	5
Family 13	Patient 17	c.634T > A	p.212Cys > Ser	4	3 and 4
Family 14	Patient 18	c.665G > A	p.222Cys > Tyr	4	3 and 4
Family 15	Patient 19	c.3058G > C	p.1020Ala > Pro	19	2,3, 5, 6, 11, 18 and 19
Family 16	Patient 20	c.457C > T	p.153Arg > Cys	4	3 and 4
Family 17	Patient 21	c.457C > T	p.153Arg > Cys	4	3,4 and 19
Family 18	Patient 22	c.1735T > C	p.579Cys > Arg	11	Information not available
Family 19	Patient 23	c.457C > T	p.153Arg > Cys	4	Information not available
Family 20	Patient 24	c.457C > T	p.153Arg > Cys	4	3 and 4
Family 21	Patient 25	c.434C > G	p.145Ser > Cys	4	3, 4 and 19
Family 22	Patient 26	c.397C > T	p.133Arg > Cys	4	3 and 4

A positive family history was documented in 19 patients, no family history was observed in 4 patients, and information regarding family history was not available for 2 patients.

The most common first major clinical event was ischemic stroke, which occurred in 19 patients (73%). At the time of the assessment, four individuals (15%) had not experienced any major clinical events; two of them had migraines, and the other two had psychiatric symptoms. Progressive gait disorder and encephalopathy were also identified as the first symptom in one case each. Seizures were reported by 3 (12%) individuals, and in one, it was the first primary clinical manifestation. One patient had encephalopathy as the initial clinical presentation, which was accompanied by a migraine attack, hallucinations, seizures, and a focal neurological deficit. The clinical and demographic profiles are summarized in Tables 2 and 3 respectively.

**Table 2:** Summary of clinical characteristics

Clinical features	Patients: n (%)
**All patients**	26
**Male gender**	10 (38%)
Positive family history*	19 (73%)
**Asymptomatic**	2 (8%)
**Cardinal symptoms**	24 (92%)
Migraine**	8 (31%)
Without aura	1 (13%)
With aura	6 (75%)
**TIA or stroke**	21 (81%)
Ischemic stroke	21 (100%)
Hemorrhagic stroke	1 (5%)
**Cognitive impairment**	17 (65%)
Mild cognitive impairment	11 (65%)
Dementia	6 (35%)
**Psychiatric symptoms**	**16 (61%)**
**Other neurological symptoms**	22 (92%)
Seizure	3 (12%)
Encephalopathy	1 (4%)
Pseudobulbar palsy	6 (23%)
Pyramidal signs	22 (85%)
Apraxic gait	6 (23%)
Bulbar signs	7 (27%)
**Vascular risk factors**	
No cardiovascular risk factors	8 (31%)
Past or current alcohol consumption*	3 (12%)
Past or current smoker*	6 (23%)
Dyslipidemia**	8 (31%)
Diabetes mellitus**	1(4%)
Hypertension**	10 (38%)
**Disability**	
mRS: 0-2	17 (65%)
mRS: 3-5	9 (35%)
**Antiaggregation therapy**	14 (54%)

Abbreviations: mRS, modified Rankin scale; TIA, transient ischemic attack.

Notes: *Information not available for two patients; **information was not available for one patient.

**Table 3:** Age of onset according to the first major clinical symptom

First major clinical symptom	Patients: n (%)	Mean age (years)
All patients	26	45
No major clinical symptom	4 (15%)	41
Transient ischemic attack/Stroke	19 (73%)	44.2
Encephalopathy	1 (4%)	51
Seizure	1 (4%)	68
Progressive gait disorder	1 (4%)	57

The neurological examination was unremarkable in 4 patients; 13 patients were clinically diagnosed with lacunar syndrome; 6 patients had apraxic gait with or without pyramidal/bulbar signs; and 2 patients had pyramidalism and sensitive alterations in all 4 limbs. For 1 patient, information on the neurological examination was not available.

All stroke patients had at least one ischemic event, and a simultaneous hemorrhagic stroke was reported in 1 subject. All ischemic strokes were related to the cerebral small vessel pathology. The mean age at the onset of ischemic episodes was of 45 years, with a broad range of 25 to 68 years).

The second most common cardinal symptom was mild cognitive impairment (MCI), which was present in 17 patients (65%), and was described as a multi-domain MCI in 11, and dementia in 6 subjects. All patients with dementia had apraxic gait on the neurological examination. Psychiatric symptoms were observed in 16 patients (61%). Migraines were present in 8 (31%) patients, and 6 (75%) of them had aura attacks.

Prior to admission, CADASIL had not been diagnosed in 13 patients (50%). A total of 8 individuals had received a diagnosis of stroke or dementia, but a hereditary disorder was not considered, and 4 patients were first-degree relatives of other affected subjects. In addition, 5 patients had an initial alternative diagnosis, including multiple sclerosis (3 cases), autoimmune encephalopathy, and polyneuropathy (1 case). A detailed clinical description of all individuals is shown in Supplementary Material Table S1 .

Brain MRIs were not available for 4 patients. In addition, susceptibility-weighted imaging (SWI) sequences were not performed in 2 cases; therefore, hemorrhage assessment was not possible for these patients. All radiologic aspects of the patients are summarized in Table 4 .

**Table 4:** Brain MRI findings overview

	SWI, T2* or b0		T2 or FLAIR					T2 or FLAIR							
	Intracerebral hemorrhage (n)		Lacunar stroke image					White matter hyperintensities							
Patient	Microbleeds	Larger hemorrhage	BG	FPM	EC	IC	Pons	TL	PV	EC	IC	EC > IC	BT	FAZEKAS score	SVD score
Patient 1	17	0	+	+	−	−	+	+	+	+	+	+	+	1	3
Patient 2	0	0	−	−	−	−	−	+	−	+	−	+	+	1	1
Patient 3	N/A		N/A					N/A						N/A	N/A
Patient 4	0	0	−	−	−	−	−	+	−	−	−	−	−	0	0
Patient 5	0	0	+	+	−	−	+	+	+	−	+	−	+	3	2
Patient 6	0	0	+	−	−	−	−	+	+	+	+	+	+	3	2
Patient 7	0	0	−	−	−	−	−	+	+	−	−	−	−	2	1
Patient 8	N/A		N/A					N/A						N/A	N/A
Patient 9	0	0	−	−	−	−	−	−	−	−	−	−	−	2	1
Patient 10	15	0	+	+	−	+	+	+	+	−	−	−	−	2	3
Patient 11	N/A		N/A					N/A						N/A	N/A
Patient 12	28	1	+	+	+	−	+	−	+	+	+	+	+	3	4
Patient 13	11	0	+	−	−	−	−	+	+	+	−	+	−	1	3
Patient 14	13	0	+	−	−	+	−	+	+	−	−	−	+	2	3
Patient 15	0	0	+	+	+	−	−	+	+	+	+	+	+	1	2
Patient 16	4	0	+	+	+	+	+	+	+	+	−	+	+	3	4
Patient 17	N/A		+	−	−	−	−	+	+	+	−	+	−	2	N/A
Patient 18	0	0	+	+	+	+	+	+	+	+	+	−	+	2	3
Patient 19	13 ^§^	0	−	−	−	−	−	+	+	−	−	−	−	2	2
Patient 20	N/A		N/A					N/A						N/A	N/A
Patient 21	18	0	+	+	−	+	+	+	+	+	+	+	+	2	3
Patient 22	N/A		+	+	+	−	+	+	+	+	+	−	−	3	N/A
Patient 23	0	0	+	+	−	+	+	+	+	+	+	−	+	3	3
Patient 24	2	0	+	+	+	+	+	+	+	+	+	+	−	3	4
Patient 25	7	1	+	+	+	+	+	+	+	+	+	+	+	3	4
Patient 26	0	0	−	+	−	−	−	+	+	−	−	−	−	2	1

Abbreviations: b0 = T2 EPI (diffusion images), baseline; BG, basal ganglia; EC, external capsule; FLAIR, fluid-attenuated inversion recovery; FPM, frontal paramedian; IC, internal capsule; MRI, magnetic resonance imaging; N/A, not available; SVD, small vessel disease; SWI, susceptibility-weighted imaging; BT, brainstem; PV, periventricular white matter; TL, thalamus.

Note: ^§^ Walnut kernel microbleed sign.

In all but 2 (91%) patients with available MRI data, WMHs were found in the temporal lobe. The same findings were observed in the external capsule in 15 patients (68%). The median Fazekas score was of 2. Lacunar infarcts were observed in 18 patients (82%), and the other 4 patients without any lacunar stroke were asymptomatic.

**Figure 1:**
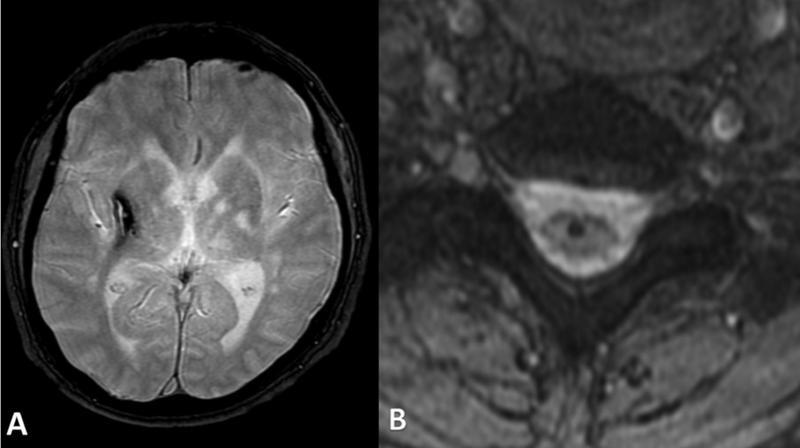
( **A** ) Axial T2*-weighted image: low signal in right basal ganglia, corresponding to hemorrhagic stroke; ( **B** ) **:** Axial T2*-weighted image: low signal in cervical spinal cord, possibly corresponding to microbleeds.

In 9 out of 20 (45%) available MRIs with SWI sequences, CMB was present. In 6 of them, the number of CMBs was greater than 10. In total, 2 patients had larger hemorrhages ( Figure 1A ), and we report 1 case of microbleeds in the cervical spinal cord ( Figure 1B ).

## DISCUSSION

We herein report the most extensive series of CADASIL patients from a Brazilian population published to date. The pathogenic mutation in exon 4 was the most common in the present study, which is in accordance with the current literature. 4 However, as aforementioned, not all suspected patients had all possible exons sequenced due to technical limitations. Therefore, there is likely an overrepresentation of individuals with exon-4 mutation, which was caused by an instrumental bias.

Ischemic stroke is the most common clinical presentation of CADASIL. We found a prevalence of 77% in the present study, similar to the frequencies reported in other cohorts, which range from 60% to 84%. 10 The exact equivalence was found for the age of onset, which usually averages approximately 41to 49 years, and, in the present study, we report an average age of onset of 44.2 years.

The migraine prevalence in CADASIL ranges from 20% to 70%, and it is less common in Asian patients. 11 The prevalence of migraines with aura in CADASIL patients is higher than expected when compared to a community cohort, reaching up to 80%. 12 In the present study, 8 patients had migraines, making this prevalence closer to that observed in Western cohorts. In addition, 6 (75%) of them had auras, which is in accordance with published data. 18

The prevalence of epilepsy has been reported to be of approximately 5% to 10% of patients. 13 In the present study, there were 3 patients (11%) with epilepsy, all of them with exon-19 mutations. However, genotype-phenotype correlations have not been well established in CADASIL. 4

An adequately-collected family health history is one of the cornerstones of CADASIL. All first- and second-degree relatives were considered. Up to 30% of the patients in one series had missing data. 14 In the present study, 27% of patients had no family history of CADASIL or any of its features. However, as the data were collected retrospectively, it was not possible to exclude the aforementioned errors. In 8 patients who suffered stroke or dementia, CADASIL or other hereditary disorders were not considered, although most of them had one or more first-or second-degree relatives with cardinal symptoms. This may indicate an unfamiliarity among clinicians and radiologists with this disease. We conjecture that good family history data are lacking in a higher proportion of these individuals, and a structured interview mentioning all cardinal symptoms might have improved the suspicion of CADASIL.

Multiple sclerosis was the initial diagnosis in 3 patients; none of them had spinal or optic nerve impairment, lesions on contrast-enhanced MRI, or CSF alterations. All patients had previously received immunomodulatory therapy with exposure to possible side effects.

In total, 1 (4%) patient had a previous diagnosis of autoimmune encephalopathy. He had an atypical presentation of CADASIL, showing an epileptic seizure as the first symptom. In addition, there was a parietal lesion with gadolinium enhancement and elevated levels of cerebrospinal fluid (CSF) protein. He was not treated with any specific immunotherapy. Alterations in the CSF have been described in the literature, and the inflammatory aspects and manifestations of CADASIL remain a matter of debate. 15 16

Only 1 patient in the sample of the present study had encephalopathy. This clinical aspect was less represented here than expected, as most known cohorts reported mean prevalence rates up to three times higher. 17 This major symptom is more common in females, it is usually the presenting feature, and the median age of onset is 40 years. 18 Typically, patients present with a range of cortical symptoms and signs, including acute confusional state, seizures, and visual hallucinations. 17 The CADASIL encephalopathy tended to be self-limited, and patients typically recovered completely within days to a few weeks without specific treatment. There is an inverse association between stroke and encephalopathy. 18 The case herein described had a typical presentation as the first major CADASIL symptom and it was associated with migraines and cortical symptoms, including hallucinations and seizures. However, she had symptoms of residual encephalopathy, a feature that was not expected and probably related to an older age at onset and a higher vascular load. Encephalopathy is not a well-known manifestation of CADASIL; therefore, this may explain the lower representation of patients in our sample. In addition, patients who only show this usually reversible feature may not seek rehabilitation centers like ours.

We also report 1 patient with a history of alcoholism and use of aspirin presenting with symptomatic intracerebral hemorrhage (ICH) as the initial manifestation of the disease. Although underestimated, symptomatic intracerebral hemorrhagic stroke is not rare in CADASIL, and its occurrence is usually associated with anti-aggregation therapy and hypertension. 19

Anti-aggregation therapy was reported in 14 patients (54%), despite not being supported by the most recent evidence. 3 However, when most of these patients were assessed, it was still a matter of debate, and, at that time, most of the authors advocated for its prescription. 1

Neuroimaging shows three types of lesions in patients with CADASIL: WMHs, lacunar infarcts, and CMBs. One of the first imaging signs of CADASIL is WMHs, which often appear before clinical onset. The anterior temporal pole and external capsules are recognized as the sites of predilection. Many publications 20 have acknowledged the anterior temporal lobe and external capsules (as sites of predilection for WMHs), which if affected, are very useful in differentiating from other forms of SVDs.

Involvement of the anterior temporal lobe and of the external capsule had approximately the same sensitivity (of approximately 90%). Nevertheless, the involvement of the temporal pole had a much higher specificity than that of external capsule involvement (100% and 45% respectively). 20 However, in Asian populations, involvement of the anterior temporal pole is less common. In our sample, WMHs in the temporal pole were more frequent than WMHs in the external capsule (91% versus 68% respectively), which is consistent with European cohorts. 1

No major clinical events were recorded for 4 patients. Brain MRI data were available for 3 of them, all of whom had WMHs in the anterior temporal pole, which reinforces the concept that these findings might be detected up to 15 years before the initial clinical manifestations. 21

The involvement of the corpus callosum, which is rare in sporadic SVD, has been described in CADASIL individuals. 21 All patients reported with a previous diagnosis of multiple sclerosis had lesions involving this site. However, none of them had the “dot-dash” appearance of the calloso-septal lesions classically seen in MS. 22

The number and volume of lacunes are MRI findings that are most strongly related to clinical deficits. 1 None of the asymptomatic patients had lacunar infarcts on MRI, and they were evidenced in all patients with dementia when the MRI was available, which could confirm this finding as a marker of severity in our sample.

Microbleeds in CADASIL are frequently observed in the thalamus and the temporal lobes. 23 Other cortical and subcortical regions have also been described as infratentorial sites. 24 We report 1 case with 3 microbleeds on cervical cord MRI. To date, we are not aware of any other reports with this finding. It is worth mentioning that it is not a usual site to look for in CADASIL. This particular patient was assessed for a differential diagnosis of myelopathy. Her first symptom was progressive gait disorder. The patient also showed pyramidal and sensitive signs in all four limbs. She was previously misdiagnosed with polyneuropathy, although none of the neurophysiological examinations supported this hypothesis. Nevertheless, the patient did not have CMBs on her brain MRI, but did have an extensive WHM, which could, in turn, explain her neurological manifestations. The significance of spinal microbleeds is uncertain; however, given the previously reported spinal commitment in the literature, this could be another aspect of the alterations in microvasculature alteration at the level of the spine.

The prevalence of CMBs in CADASIL varies across different cohort studies (25% to 87%), and is much more common in Asian countries. 23 In our sample, CMBs were found in 45% of patients with available SWI images, so they were more prevalent than in European cohorts and less prevalent than in Asian cohorts. Ethnicity might be one of the reasons for the discrepancies because Asians are more prone to ICH hemorrhage than Caucasians, Blacks, and Hispanics. 25

Furthermore, this discrepancy could also be explained by sociodemographic characteristics such as inadequate risk control and an unhealthy lifestyle, which were have been described in low- and middle-income countries. 26

The prevalence of spontaneous ICH ranges from 0.5% to 21%. We found ICH in 2 patients (10%): 1 asymptomatic and 1 symptomatic subject. A total CMB count > 10, the presence of CMBs in the brain stem, and SVD scores have been associated with the presence of ICH. 22 The 2 patients with ICH in the present study had high SVD scores, but 1 patient (50%) had a total CMB count < 10. A total of 8 patients without ICH (33%) had a total CMB count > 10, indicating that it is a weak biomarker in our population.

Hypertension and antithrombotic agents are the most critical risk factors for ICH in patients with CADASIL. One patient had hypertension and the other was taking aspirin. These data have clinical implications as a more intensive target of systolic and diastolic blood pressure < 120/80 mmHg should be considered in patients with CADASIL with ICH, and caution should be taken regarding the prescription of antiplatelet therapy. 19

Despite the data gathered, the present study had the following limitations: 1) the retrospective design; 2) the possible selection bias due to it being conducted in a quaternary rehabilitation center; 3) the instrumental bias, as many patients only had exons 3 and 4 analyzed; and 4) the neuroimaging was not performed in the same service in all cases.

In conclusion, to date, the present study is the most extensive series of CADASIL patients from a Brazilian population. Despite being the most common hereditary vascular disease, it remains largely unknown among general practitioners and radiologists.

Most of our clinical and epidemiological data are in accordance with those of European cohorts, except for the microbleeds and hemorrhagic strokes, for our results are closer to those of Asian cohorts. Brazil has one of the most heterogeneous genetic constitutions in the world, with a trihybrid-based composition of European, Native American and African ancestries. 7 A better understanding of the clinical and genetic profiles of our population is paramount.

To the best of our knowledge, this is the first report of microbleeds in the spinal cord in a patient with CADASIL.
