# Supplementary material for: Clinical and epidemiological profiles from a case series of 26 Brazilian CADASIL patients
Source: Arq Neuropsiquiatr. 2023 May 8;81(5):417–25. doi: 10.1055/s-0042-1758756 (PMC10232034; doi:10.1055/s-0042-1758756)
Supplement: Supplementary file 1 — Supplementary Material [file 10-1055-s-0042-1758756-s210480.pdf]

Supplementary Material Table S1 Summary of clinical characteristics

Patients	Sex	Age at onset (years)	First major event	CV event	Migraine	Cognition	Psychiatric disorder	Seizures	mRS
Patient 1	F	68	Encephalopathy	AIS	With aura	MCI	Present	Present	3
Patient 2	M	–	None	None	With aura	Normal	Absent	Absent	0
Patient 3	F	–	None	None	Absent	Normal	Absent	Absent	0
Patient 4	F	–	None	None	Absent	Normal	Present	Absent	0
Patient 5	F	46	AIS	AIS	Absent	Dementia	Absent	Absent	5
Patient 6	F	57	Progressive gait abormality	None	Absent	Normal	Absent	Absent	2
Patient 7	F	–	None	None	Without aura	Normal	Absent	Absent	0
Patient 8	M	56	AIS	AIS	Absent	Dementia	Present	Absent	4
Patient 9	F	36	AIS	AIS	Without aura	MCI	Absent	Absent	1
Patient 10	F	46	AIS	AIS	With aura	Normal	Present	Absent	2
Patient 11	M	58	AIS	AIS	Absent	Dementia	Absent	Absent	4
Patient 12	F	43	AIS	AIS	Absent	Dementia	Absent	Absent	4
Patient 13	F	40	AIS	AIS	Absent	Normal	Present	Absent	2
Patient 14	M	57	AIS	AIS	Absent	MCI	Present	Absent	2
Patient 15	M	25	AIS	AIS	With aura	MCI	Present	Absent	2
Patient 16	F	40	AIS	AIS	With aura	MCI	Present	Absent	2
Patient 17	M	42	AIS	AIS	Absent	Normal	Present	Absent	N/A
Patient 18	F	35	AIS	AIS	With aura	MCI	Present	Absent	2
Patient 19	M	51	Seizure	AIS	Absent	MCI	Present	Present	2
Patient 20	M	41	AIS	AIS	Absent	Dementia	Present	Absent	5
Patient 21	M	52	AIS	AIS	Absent	MCI	Present	Present	2
Patient 22	F	50	AIS	AIS	Absent	Dementia	Absent	Absent	5
Patient 23	F	35	AIS	AIS	Absent	MCI	Present	Absent	2
Patient 24	F	56	AIS	AIS	Absent	MCI	Absent	Absent	3
Patient 25	M	40	AIS	AIS/HS	Absent	MCI	Present	Absent	2
Patient 26	F	43	AIS	AIS	Absent	Normal	Present	Absent	2

Abbreviations: AIS, acute ischemic stroke; CV, cerebrovascular; HS, hemorrhagic stroke; F, female; mRS, modified Rankin scale; M, male; MCI, mild cognitive impairment; N/A, not available.

 © 2023. Academia Brasileira de Neurologia. All rights reserved.
